# Targeted Screening for Cancer: Learnings and Applicability to Melanoma: A Scoping Review

**DOI:** 10.3390/jpm14080863

**Published:** 2024-08-14

**Authors:** Lejie Zheng, Amelia K. Smit, Anne E. Cust, Monika Janda

**Affiliations:** 1 Centre for Health Services Research, The University of Queensland, St. Lucia, QLD 4067, Australia; lejie.zheng@uq.edu.au; 2 The Daffodil Centre, The University of Sydney, a Joint Venture with Cancer Council NSW, Sydney, NSW 2006, Australia; amelia.smit@sydney.edu.au (A.K.S.); anne.cust@sydney.edu.au (A.E.C.); 3 Melanoma Institute Australia, The University of Sydney, Sydney, NSW 2065, Australia

**Keywords:** personalized screening, cancer prevention, melanoma, scoping review

## Abstract

This scoping review aims to systematically gather evidence from personalized cancer-screening studies across various cancers, summarize key components and outcomes, and provide implications for a future personalized melanoma-screening strategy. Peer-reviewed articles and clinical trial databases were searched for, with restrictions on language and publication date. Sixteen distinct studies were identified and included in this review. The studies’ results were synthesized according to key components, including risk assessment, risk thresholds, screening pathways, and primary outcomes of interest. Studies most frequently reported about breast cancers (*n* = 7), followed by colorectal (*n* = 5), prostate (*n* = 2), lung (*n* = 1), and ovarian cancers (*n* = 1). The identified screening programs were evaluated predominately in Europe (*n* = 6) and North America (*n* = 4). The studies employed multiple different risk assessment tools, screening schedules, and outcome measurements, with few consistent approaches identified across the studies. The benefit–harm assessment of each proposed personalized screening program indicated that the majority were feasible and effective. The establishment of a personalized screening program is complex, but results of the reviewed studies indicate that it is feasible, can improve participation rates, and screening outcomes. While the review primarily examines screening programs for cancers other than melanoma, the insights can be used to inform the development of a personalized melanoma screening strategy.

## 1. Introduction

Cancer was responsible for nearly one-sixth of global mortality, with an estimated 10 million deaths occurring in 2020 [1]. Early cancer detection through population-based screening programs has already resulted in significant improvements in survival rates for several cancers, including breast, cervical, and colorectal [2]. Although there are many benefits for reducing cancer incidence and mortality, population-based cancer-screening programs are also resource-intensive and may lead to overdiagnosis. This phenomenon pertains to individuals who, if left untreated, would not have been impacted by the detected cancer [3]. Overtreatment has also been reported as a result of false positive screening examinations [4,5]. Currently, most organized cancer-screening programs are based on fixed eligibility requirements such as age and sex. In a shift away from the ‘one size fits all’ approach, interest in personalized screening is growing [6,7].

Personalized screening approaches take into account individuals’ risk-factors to assess eligibility and determine screening frequency, such as genetic risk, family history and lifestyle factors [8,9]. One recent example is a lung-cancer-screening trial conducted in the United States, which was introduced only for people who have a strong smoking history [10]. Risk assessment at population level is thought to enable the identification and targeting of groups at elevated risk who stand to benefit the most from screening and may reduce or not offer screening for those who will benefit the least and for whom the benefit/risk ratio of screening is therefore worse [7]. Personalized screening is potentially more cost-effective and may require less healthcare resources than current population-based approaches [8,9,11]. As it would increase the prevalence of true disease in the screened group, it could minimize the risk of overdiagnosis and unnecessary treatments [5,9,12].

Many countries have well-established, organized, population-based screening programs in place for the early detection of breast [13], cervical [14], and colorectal cancers [15]. Although melanoma is less common than other types of skin cancer, it is the most serious. While some European countries, such as Germany and France [16,17], have implemented national guidance on melanoma screening by physicians in a legal framework, these guidelines do not qualify as personalized screening due to a lack of risk assessment for screening eligibility and/or the absence of tailored screening frequencies based on individual risk [18]. Consequently, there is a lack of evidence of effectiveness and cost-effectiveness in support of personalized melanoma screening [19]. Early detection of melanoma, along with keratinocyte cancers such as basal cell carcinoma and squamous cell carcinoma, is therefore incidental and usually initiated by the patient or a health professional [20]. The limitations of opportunistic early detection are reflected in inequitable melanoma outcomes among the community, with better outcomes for higher socioeconomic groups and those living near metropolitan centers [21,22,23]. In Australia, treatment for melanoma and other keratinocyte cancers combined already confer the highest healthcare cost burden compared to other cancers [24]. The burden from melanoma and other skin cancers is projected to increase substantially over the next two decades due to aging of the population, and increasing ultraviolet radiation exposure due to climate change [25], which could further increase the risk of overdiagnosis and overtreatment [26].

International recommendations [19,27] and Australian clinical practice guidelines already support personalized approaches to melanoma early detection, recommending clinicians identify people at high risk and conduct a skin examination, but there is limited evidence on how this should be implemented. A randomized, controlled trial of a personalized melanoma-screening program could help determine the benefits, costs, harms, and potential implementation strategy [28,29]. However, there are several complexities to establishing personalized screening programs that require decisions about the best approach to risk assessment, risk communication, risk thresholds, screening examination, and screening intervals.

To explore the feasibility and implementation strategies of personalized screening, we conducted a scoping review of relevant risk-based studies across various screen-detectable cancers. The World Health Organization’s principles and practice of screening for disease served as the foundational framework for this scoping review [30]. By leveraging insights and lessons learned from research in other cancer types, there is a significant opportunity to inform the design of a potential future personalized melanoma-screening program.

## 2. Methods

**Figure 1:**
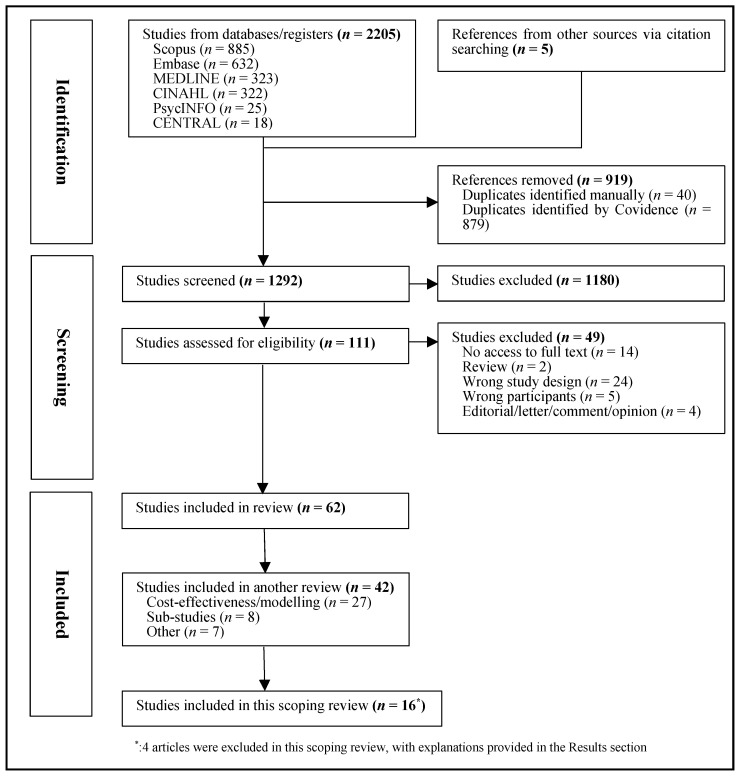
Flowchart of selection of studies.

This review was conducted according to the PRISMA extension for scoping reviews and the Population/Concept/Context (PCC) Framework [31] and registered on the Open Science Framework (registration DOI: https://doi.org/10.17605/OSF.IO/FC8W4, accessed on 22 May 2024). The search process is displayed in Figure 1.

### 2.1. Data Source

Electronic databases, including Medline, Embase, Cochrane central, PsychINFO, CINAHL, Scopus, ANZCTR, and clinicaltrials.gov, were systematically searched. Eligible articles for this scoping review included those published in English from January 2013 to May 2023.

### 2.2. Study Selection

English language reports published in peer-reviewed sources or listed on clinical trials databases were included, in accordance with specific inclusion and exclusion criteria. The search strategy, terms, and exclusion/inclusion criteria were developed in consultation with an experienced librarian at the University of Sydney. The detailed search strings for each database are provided in the Supplementary Materials.

Studies were deemed eligible if they met all of the following criteria:

- Participants from the adult general population;
- Studies on personalized cancer-screening programs, where screening was tailored according to personal risk (beyond age or sex alone);
- Focus on cancer(s);
- January 2013 to May 2023, to focus on contemporary screening programs;
- Research in humans.

The studies were excluded if they met any of the following criteria:

- Written in a language other than English;
- General screening (not risk-based) studies;
- Reviews, letters, editorials;
- Evaluations of hypothetical personalized cancer screening.

### 2.3. Data Extraction

Data extraction was performed independently by two reviewers, and discrepancies were addressed through discussion. Key variables from each paper were compiled in a data extraction framework and included the following: author, year, country, study design, primary aim, cancer type, study period, outcomes and measures, risk assessment, risk thresholds, screening intervals, participants characteristics, outcomes measured, and results.

### 2.4. Quality Assessment

Quality assessment was carried out using the Cochrane Risk of Bias Tool for Randomized Controlled Trials [32] and the Risk Of Bias In Non-randomized Studies—of Interventions (ROBINS-I) [33] for non-randomized studies.

## 3. Results

A total of 2205 records were identified. Five additional papers were found through hand searching. After removing 919 duplicates, 1292 articles underwent title and abstract screening. Subsequently, 111 were selected for full-text review, with 49 being excluded (detailed reasons are provided in the Supplementary Materials). Reasons for exclusion were no access to full text (*n* = 14), review (*n* = 2), wrong study design (*n* = 24), wrong participants (*n* = 5), and editorials/ letter/ comments/ opinion (*n* = 4) (Figure 1).

This broader search retrieved 62 articles. Of these, 42 studies were focused on modeling or health economics (*n* = 27), reported on screening sub-studies (*n* = 8), or were categorized as ‘other’ (*n* = 7), and will be reported in a separate review. For this scoping review, 20 studies were selected, including 16 distinct personalized cancer-screening studies and 4 study protocols that focused on the evaluation of personalized cancer screening. Due to overlap with the identified results articles, the 4 protocol papers were excluded from this scoping review. Thus, the present review focuses on the 16 articles that reported evaluations of personalized cancer-screening implementation, including the key study components and outcomes (Figure 1). Overall, the studies included were of good quality. The overall risk of bias assessment for each study is provided in Supplementary Materials.

### 3.1. Key Characteristics of Eligible Studies

The key characteristics of the 16 studies are detailed in Table 1. Most studies were published from 2020 onwards. The majority of study designs were randomized controlled trials (*n* = 8) [11,34,35,36,37,38,39,40], followed by cohort studies (*n* = 2) [41,42]. Others included a non-randomized controlled trial [43], feasibility [44] and proof-of-concept [45], mixed-methods [46], pre–post implementation [47], and focus group [48] studies.

### 3.2. Cancer Types

The included studies mainly focused on breast cancer (*n* = 7) [38,41,42,43,45,46,48], colorectal cancer (*n* = 5) [11,34,35,39,40], prostate cancer (*n* = 2) [36,47], lung cancer (*n* = 1) [37], and ovarian cancer (*n* = 1) [44].

### 3.3. Study Locations

The study locations included six in European countries [36,37,43,44,45,48], four in North America [35,38,44,46], three in Australia [11,39,40], and three in Asian countries [34,41,42].

### 3.4. Participants

The study sample sizes varied greatly, spanning from over 1 million participants [42] to as few as slightly over a hundred [44]. Furthermore, studies varied widely in eligibility criteria, risk assessment tools, risk thresholds, and other key elements. For example, the cancer-screening programs ranged widely in terms of the age ranges considered for participation. In the context of breast cancer, most studies targeted unaffected women between 40 and 70 years [38,42,45,46,48], while Gareth Evans et al. specifically concentrated on women who were approaching the age of 60 years [43]. For colorectal cancer screening, adults 25 to 75 years were included [11,34,35,39,40]. Prostate cancer screening included males after reaching adulthood [36,47], while ovarian cancer screening recruited females once they turned 18 [44].

### 3.5. Risk Assessments

Tools used for risk assessment in each study are presented in Table 2. Most studies used multiple data sources to predict an individual’s risk of developing cancer. Among the combinations with different approaches found in the 16 studies, eight incorporated self-reported questionnaires, blood, and/or saliva tests, and validated risk prediction model [37,38,41,43,45,46,48]. Three studies combined questionnaires with blood/saliva test results [36,40,47]. One study combined questionnaire data with polygenic risk score and a risk prediction model [44]. Five studies relied solely on self-reported questionnaire data for risk estimation [11,34,35,39,42].

### 3.6. Risk Thresholds and Proposed Pathways

The risk thresholds and screening intervals are summarized for each cancer type in Table 3. There was no consistency in the risk thresholds or number of groupings used in the included studies. The majority categorized potential screening participants into two (high and low) risk groups [11,34,35,37,38,41,42,47] or three groups [36,38,39,40,44,45,46], while two studies incorporated four risk groups [43,48]. Several variations were also found with regard to the proposed screening intervals. Most screening intervals were based on a combination of participant’s age, family history, and risk level, resulting in a range of two to eight proposed screening pathways. Four studies did not propose pathway recommendations [35,36,44].

In a mixed-methods study from Brooks et al. [46], three risk categories were defined based on 10-year/remaining lifetime risk, namely less than 15% as average risk, 15–25% as higher than average, and 25% and above as high risk. The corresponding screening pathways were determined based on both participants age and their estimated risk levels. For those categorized as higher than average risk, apart from age and risk thresholds, screening pathways were also determined according to their residence location. For instance, participants in the Province of Ontario underwent an annual mammogram, while those residing in the Province of Québec followed a mammogram screening schedule of everyone to two years, with the possibility of incorporating ultrasound assessments if breast density exceeded 75%. In a separate study conducted in the United Kingdom [48], risk thresholds were classified based on 10-year risk, including low risk (≤1.5%), average risk (1.5–4.99%), moderate risk (5–7.99%), and high risk (≥8%). In contrast to the approach of Brooks et al. [46], the screening pathways in this study were solely dependent upon the assigned risk categories. For participants categorized as low to moderate risk, their screening frequency was every 3 years, while those in the high-risk group should follow an 18-month screening schedule. Detailed information specific to each study is available in Table 3.

### 3.7. Key Components of Eligible Studies

The key components of the reviewed studies extracted include their primary outcomes, measurement methodologies, and main findings. Out of 16 studies, the majority directed their focus towards the practical consideration inherent in the newly proposed personalized screening approach. Two studies were not completed at the time of conducting this scoping review [38,46], and two papers only presented study protocols without any released results [40,41]. The primary outcomes in the selected studies are summarized in line with the World Health Organization (WHO) Wilson Framework (condition, test, treatment, and screening program) [30] and displayed in Table 4.

#### 3.7.1. Screening Process and Evaluation

Four studies conducted an analysis of recruitment outcomes by assessing the acceptability and/or feasibility of the suggested screening approach [37,41,44,45]. Six studies evaluated screening by reporting the participation rate of the targeted population who underwent screening over subsequent follow-up examinations [11,36,39,40,43,47]. Three studies reported the percentage of the screened population that required further assessment [34,38,42]. Another three studies focused on longer term outcome measurements, including morbidity [38] and mortality [38,42]. Two studies addressed other aspects within the screening process; one of them compared concordance between participants’ preferences and ordered screening test [35], while the other explored the adoption of healthy behaviors following participation in a screening program [48].

#### 3.7.2. Main Outcomes of Screening

The primary outcomes of this scoping review include the potential benefits and harms associated with each screening program, categorized by cancer type and summarized in Table 5. The majority of studies concluded that the evaluated screening strategies were feasible and cost-effective. Potential benefits included improved cancer knowledge [36], positive attitudes and screening examination uptake [11,35,39,43,44,45,47,48], a higher cancer detection rate at an early stage [37], reduction in cancer-related deaths [42], and cost-effectiveness [34,37].

The most frequently identified harms were false positive screening results and overdiagnosis [37,42,45,47,48]. Other potential harms included higher costs [34,39] and failure to align with participants’ preferences in a clinical setting [35].

Potential benefts and harms were unassessable for the studies still underway [38,46] or were presented in protocol format only [40,41].

#### 3.7.3. Results by Cancer Types

In the context of breast cancer, uptake of the personalized screening program was acceptable [43], with positive feedback received from the participants [45,48], and the proposed personalized screening approach resulted in an increased cancer detection rate among all groups [42].

For colorectal cancer, the implementation of the suggested personalized screening approaches resulted in a higher participation rate for all screening groups [11,39] or high-risk groups only [39]. Chen et al. found that the proposed risk-based screening led to an increased cancer detection rate while requiring a reduced number of colonoscopies to be performed [34].

For the two prostate-cancer-screening studies, both studies observed an increased proportion of men adhering to the risk-appropriate recommended prostate-specific antigen (PSA) test schedules [36,47].

Regarding other cancer types, the use of a lung cancer risk prediction model was tested and found to be effective for identifying high-risk individuals who would be suitable for lung cancer screening [37], while a personalized ovarian cancer-screening program was appraised as acceptable and satisfactory by the participants, as indicated by validated questionnaires [44].

## 4. Discussion

There has been increasing interest in personalized cancer screening over the past decade, alongside accumulating evidence on its potential advantages compared to population-based cancer screening which may be unnecessary for those at low risk, and not intensive enough for those at high or very high risk. In this scoping review, we identified several key components and outcomes that may assist in the design and evaluation of future personalized screening studies and programs for implementation in melanoma settings. Clinical guidelines already recommend personalized approaches to early detection of melanoma, yet evidence on the benefits and harms of screening is currently insufficient. The targeted screening interventions reviewed 16 studies reported on personal risk-assessment design, tailored follow-up intervals for future check-ups, and primary outcomes of interest across multiple cancer types. While none specially targeted melanoma, these studies explored important aspects of the screening process that could be applicable to personalized melanoma screening. These findings are discussed within the framework of the WHO screening guidelines.

Prior investigations into cancer screening across various cancer types have been included into this review. While population-based screening programs for ovarian cancer have not been approved globally, this scoping review included the feasibility study for this cancer type, given the ongoing efforts in this area [49]. The screening studies included in this review demonstrated participation rates of over 50%, which were deemed acceptable [11,36,39,40,43,47]. The WHO guidelines highlight the importance of participant engagement to maximize recruitment to screening, as high participation rates form the foundation for effective screening programs. Several of the reviewed studies examined whether personalized approaches improved the acceptability and/or feasibility of cancer screening [37,41,44,45]. These studies showed positive attitudes towards the personalized cancer screening programs implemented, which further indicates that personalized screening programs may engage those that benefit the most from screening more effectively. Although none of the included studies specifically addressed melanoma screening, it is worthwhile to examine shared characteristics in the design of personalized screening programs, such as personalized cut-off scores for different demographic cohorts, proposed screening pathways, and suggested follow-up intervals. Such analysis would further enrich the groundwork for designing future melanoma screening pilot studies. We found that various risk assessment strategies were incorporated in the design of the personalized screening programs to enable more accurate risk assessment. The commonly self-reported risk factors included in the risk tools were age, gender, race, personal cancer history, family history of cancer, and lifestyle factors, which were also relevant for defining melanoma high-risk individuals. Moreover, some studies also included the assessment of genetic- or biomarkers from blood or saliva samples. Currently, in the context of melanoma, several self-reported risk factor online tools are already freely accessible to both health professionals and the public [50,51], which calculate personal melanoma risk based on general risk factors plus melanoma specific ones such as phenotypic characteristics (hair, skin, eye color), sun or sunbed exposure, and melanocytic nevus (moles) counts. Scientific literature has highlighted concerns regarding the lack of validation in current melanoma risk prediction models [52,53]. This issue must be addressed when designing personalized screening strategies, as a validated risk-prediction model is essential for ensuring effective and valid personalized melanoma screening. Genomic risk factors, such as polygenic scores, are also related to risk of many cancers, including melanoma, and are anticipated to be incorporated into risk prediction tools more commonly [54,55]. In addition, our search showed that ten studies included polygenic scores in their risk assessment process, suggesting a necessity to further explore whether the addition of genomic information improves risk assessment enough to be incorporated in personalized screening programs, as this is more complex and costly compared to self-reported risk only.

Two studies in this review reported an increased cancer detection rate [34,42]. While this can be seen as a benefit, it could also be a result of length–time bias and also raises concerns about potential overdiagnosis and overtreatment [37,42,45,47,48]. Despite the demonstrated benefits of nationwide mammographic screening programs in reducing breast cancer deaths reported in many countries [56,57,58,59,60,61], the overall effectiveness of screening is still debated due to evidence of potentially unnecessary treatments. Overdiagnosis is a major concern for melanoma screening, as incidence rates increased substantially over the past decade without concomitant decreases in mortality [62]. In Australia, 54% of all melanomas (including melanoma in situ) and 15% of invasive melanomas are estimated to be over-diagnosed [26] in the absence of a formal dedicated screening program [63].

The ultimate goal of cancer-screening programs is to reduce mortality and morbidity [29]. In the context of melanoma, no randomized controlled trial has evaluated the screening impact on melanoma mortality, although observational studies have suggested that having a screening examination during the past 3 years reduces the thickness of melanoma [19,22]. Based on the reviewed personalized screening programs in other cancers, morbidity and melanoma thickness might be a suitable primary parameter, given the low overall mortality risk from melanoma, particularly with advancements in targeted and immunotherapy treatments [64,65]. In addition, other considerations for implementing a personalized melanoma screening program in Australia should include medical intervention practices, healthcare accessibility across different regions, costs, and individual quality of life. Potential parameters in this context include the need for adjuvant or neoadjuvant treatment, individual’s quality of life, healthcare expenditure, or equitable access or outcomes. Given the variability in melanoma risk among individuals, influenced by factors such as age, gender, race, personal cancer history, family history of skin cancer, lifestyle factors, and phenotypic characteristics, the frequency of evaluations should be tailored to individual risk levels.

### Strengths and Limitations

Evidence collected from this scoping review encompassed multiple cancer types, research methods, study locations, and provided up-to-date evidence. In addition, this review summarized a wide variety of outcomes assessed, ranging from knowledge and attitudes to mortality. Given our particular interest in melanoma screening, it was a limitation that no studies associated with melanoma or skin cancer were discovered in the search; however, insights for melanoma screening can be gained from personalized screening studies and programs for other cancer types. Indeed, numerous parallels existed in the design of cancer-screening initiatives, including the essential elements considered for high-risk groups, primary outcomes of interest, and strategies for participants recruitment. Second, because of the nature of a scoping review, we included studies with diverse methodologies, populations, and primary outcomes. This heterogeneity can make it challenging to identify patterns across different studies. Furthermore, the search was restricted to English peer-reviewed articles, which potentially prevented us from exploring non-English datasets or screening programs in regions where English is not the official language.

## 5. Conclusions

Given the limited evidence available on personalized melanoma screening, this scoping review summarizes the key components and outcomes that personalized screening studies have considered across different cancer types over the past decade. In addition to traditional screening outcomes such as cancer mortality and incidence, acceptability and feasibility to recruit were also identified as important aspects to ensure optimal screening-program effectiveness. In addition, measurement of potential harms such as overdiagnosis and overtreatment, and the balance of benefits and harms across different risk groups, are essential considerations for any screening program going forward, particularly in the context of melanoma. The sensitivity, specificity, and positive predictive value of the proposed screening method are vital factors to ensure the program’s effectiveness in reducing melanoma-related morbidity and mortality. Insights from this review can assist researchers, clinicians, and policymakers in designing new studies and programs for personalized melanoma screening.

## Figures and Tables

**Table 1:** Key characteristics of Selected studies.

Cancer(s)	Author (Year)	Location	Period	Study Design	Participants		Risk Thresholds	Screening Pathways
					Sample	Size		
Breast	Brooks (2021) [46]	Canada	2021–2022	Mixed-methods	Unaffected women; 40–69 years	5000	10-year risk corresponding to remaining lifetime risk: Average: <15%; Higher than average: 15–25%; High: ≥25%	Average: 40–49 years: no regular screening with mammogram; 50–69 years: screen with a mammogram/2 years. Higher than average: - Ontario 40–49 years: screen with mammogram/year; 50–69 years: screen with mammogram/year; - Quebec Screen with mammogram/1–2 years, ultrasound considered if breast density is >75%; High: 40–69 years: screen with mammogram and ^†^ MRI/year.
	Esserman (2017) [38]	United States	2016–2021	** RCT	Unaffected women; 40–74 years	100,000	5-y absolute risk: <1.3%; 1.3–6%; ≥6%	50 years: Digital mammography/2 years; 40–49 years: Screening is recommended when their 5-year risk equals or exceeds that of the average woman aged 50 years. Screen/2 year, except women with extremely dense breasts, who will be offered annual screening.
	Evans (2023) [43]	United Kingdom	2019–2021	Non-RCT	Biologically female reach 60 years;	2472	Below average risk: 10 years < 2%; Average risk: 2% ≤ 10 years < 5%; Moderate risk: 5% ≤ 10 years < 8%; High risk: 10-years ≥ 8%	No screening intervals specified; participants were followed at baseline, 3 months, and 6 months
	Laza-Vasquez (2022) [45]	Spain	2019–2021	Single-arm proof-of-concept study	Women; 40–50 years;	387	Absolute risk of breast cancer at 5 years: <0.99%; 0.99–1.16%; >1.16%	40–44 years: <0.99%—watch and wait; 0.99–1.16%—biennial; >1.16%—annual. 45–48 years: <0.99%—watch and wait; 0.99–1.19%—biennial; >1.19%—annual. 49–50 years: <0.8%—triennial; 0.8–1.19%—biennial; >1.19%—annual. 40–50 years: >6%: referral to hospital breast unit and or genetic counseling
	Liu (2022) [41]	Singapore	2021	Cohort study	Women; 35–59 years; No cancer history	3500	5-years absolute risk above 3% as a threshold	Current national guidelines: 35–39 years: no recommendation; 40–49 years: mammography screening/year; 50–59 years: mammography screening/2 years; In this study, women identified to be above average in breast cancer risk are referred to breast specialists at designated study sites, in addition to prevailing guidelines.
	Rainey (2022) [48]	United Kingdom	2014–2019	Focus group	Unaffected women; 50–70 years;	325	10-year risk: Low risk: ≤1.5%; Average risk: 1.5–4.99%; Moderate risk: 5–7.99%; High risk: ≥8%	Low risk: screening/3 years; Average risk: screening/3 years; Moderate risk: screening/3 years; High risk: screening/18 m
	Yen (2016) [42]	Taiwan	1999–2009	Cohort study	Asymptomatic women; 40–69 years	1,429,890	High risk: risk scores > median value of the underlying population	Single screening method: 1 clinical breast examination (CBE); 1 risk-based mammography; 1 universal mammography; 2 screening methods: Risk-based and universal mammography; CBE and universal mammography; CBE and risk-based mammography; 3 screening methods: Risk-based + universal mammography + CBE.
Colorectal	Chen (2023) [34]	China	2018–2021	** RCT	Unaffected; 50–74 years;	19,373	5-year risk: Low-risk: APCS < 4; High-risk: APCS ≥ 4	Arm 1 (*n* = 3883): 1-time colonoscopy; Arm 2 (*n* = 7793): annual ^††^ FIT; Arm 3 (*n* = 7697): - Low: ^††^ FIT/year for 3 years; - High: colonoscopy/year for 3 years.
	Emery (2023) [11]	Australia	2017–2018	** RCT	50–74 years;	734	Intervention group determined by CRISP-calculated 5-year CRC risk: 2.5% as threshold from biennial FOBT testing to 5-yearly colonoscopy; Control group determined by family history in accordance with the NHMRC-endorsed guidelines	At baseline, 1, 6, and 12 months post-randomization.
	Saya (2022) [40]	Australia	2022	** RCT	45–70 years	274	10-years risk: <1%; 1–4%; ≥4%	Risk <1%: no screening recommended; Risk 1–4%: iFOBT Risk ≥4%: colonoscopy.
	Schroy (2016) [35]	United States	2012–2014	** RCT	50–75 years; due for CRC screening in an urban safety net Hhealthcare setting	352	Low risk: cumulative scores <5; Intermediate/high risk: cumulative scores: 6–12;	No screening intervals specified.
	Trevena (2022) [39]	Australia	2012–2014	Cluster RCT	25–74 years	1495	Lifetime risk: At or slightly above average risk; Moderately increased risk; Potentially high risk.	At or slightly above average risk: ≥50 years—FOBT/2 years; Moderately increased risk: 1 colonoscopy in past 5 years if >50 years old, or if older than 10 years, earlier than youngest bowel cancer diagnosis in first-degree relatives. Potentially high risk: assessment by familial cancer service (or similar) OR colonoscopy at a frequency consistent with family history (as assessed by a panel of clinicians).
Prostate	Fredsøe (2020) [36]	Denmark	2013–2014	Cluster RCT	Men; 18–80 years; Normal PSA test result (normal PSA: <3.0 ng/mL for men below age 60; <4.0 ng/mL for men aged 60–70; <5.0 ng/mL for men aged ≥70)	5000 (actual number registered at clinicaltrials.gov)	Lifetime risk: High risk: ≥30% Normal risk: <30% Unknown risk: cannot be estimated due to missing information for family history	No screening intervals specified; participants were followed at 2 years.
	Shah (2021) [47]	United States	2016–2018	Pre-post study	Men; 40–75 years	Pre: 49,053; Post: 49,980	High risk: African-Americans, with family history of prostate cancer/abnormal genetic evaluation	40–49 years: PSA ≥ 1.5 ng/mL—refer to multidisciplinary prostate screening clinic; PSA < 1.5 ng/mL and high risk—screen/2 years; PSA < 1.5 ng/mL and average risk—resume screening at age 50; 50–69 years: PSA < 3 ng/mL—screen/2 years; PSA ≥ 3 ng/mL—refer to multidisciplinary prostate screening clinic; 70–75 years: PSA ≤ 6.5 ng/mL—refer to multidisciplinary prostate screening clinic; PSA > 6.5 ng/mL—screen/2 years.
Lung	Field (2015) [37]	United Kingdom	2011	** RCT	50–75 years; 5-year lung cancer risk of ≥5% based on the LLPv2 risk prediction model	4055	LLPv_2_ risk prediction model selects subjects with ≥5% risk of developing lung cancer in 5 years	- No nodules or category 1 (benign) nodules: no further action required; - Category 2 (small, probably benign) nodules: follow-up CT; - Category 3 (larger, potentially malignant) nodules: follow-up CT scan at 3 months and 12 months; - Category 4 (higher chance of malignancy) nodules: immediate referral to multidisciplinary team (MDT); - Nodules > 500 mm^3^ or 10 mm maximum diameter at baseline (category 4) or nodules that demonstrated growth on follow-up CT (as defined by a volume doubling time <400 days) were referred to the local MDT for further assessment.
Ovary	Gaba (2020) [44]	United Kingdom	2017	Feasibility	Women; ≥18 years; No ovarian/tubal/primary peritoneal cancer or ovarian cancer susceptibility genes	123	Lifetime risk: Low risk: <5%; Intermediate risk: 5–10%; High risk: ≥10%	No screening intervals specified;

** RCT: randomized controlled trial; ^†^ MRI: magnetic resonance imaging; ^††^ FIT: fecal immunochemical test.

**Table 2:** A summary of the risk assessment approaches employed in the selected studies (by cancer type).

Cancer (s)	Author (Year)	Questionnaire	Imaging, Blood, or Saliva Test	Polygenic Score	Model	Combinations
Breast	Brooks (2021) [46]	✓	✓ (Mammographic density)	✓ Single-nucleotide polymorphisms	✓ BOADICEA model	✓
	Esserman (2017) [38]	✓	✓	✓	✓ Breast Cancer Surveillance Consortium (BCSC) model	✓
	Evans (2023) [43]	✓	✓ (Mammographic density)	✓ Single-nucleotide polymorphisms	✓ Tyrer–Cuzick risk model	✓
	Laza-Vasquez (2022) [45]	✓	✓ (Mammographic density)	✓ Single-nucleotide polymorphisms	✓ Breast Cancer Surveillance Consortium (BCSC) model V2.0	✓
	Liu (2022) [41]	✓	✓ (Mammographic density)	✓ Single-nucleotide polymorphisms	✓ BOADICEA model Gail model	✓
	Rainey (2022) [48]	✓	✓ (Mammographic density)	✓ Single-nucleotide polymorphisms	✓ Tyrer– Cuzick (TC) risk prediction model	✓
	Yen (2016) [42]	✓	X	X	X	X
Colorectal	Chen (2023) [34]	✓ [Asia-Pacific Colorectal Screening (APCS) score]	X	X	X	X
	Emery (2023) [11]	✓ [Colorectal cancer RISk Prediction (CRISP) risk tool]	X	X	X	X
	Saya (2022) [40]	✓	X	✓ Single-nucleotide polymorphisms	X	✓
	Schroy (2016) [35]	✓ (6-item risk assessment tool)	X	X	X	X
	Trevena (2022) [39]	✓ [Colorectal cancer (CRC risk calculator)]	X	X	X	X
Prostate	Fredsøe (2020) [36]	✓)	✓ (PSA test)	✓ Single-nucleotide polymorphisms	X	✓
	Shah (2021) [47]	✓	✓ (PSA test)	X	X	✓
Lung	Field (2015) [37]	✓	✓ [Computed tomography (CT) scan]	X	✓ LLPv_2_ risk prediction model	✓
Ovary	Gaba (2020) [44]	✓	X	✓ Single-nucleotide polymorphisms	✓ (Epidemiological/hormonal/reproductive data combined with genetic information)	✓

✓: if a certain approach was incorporated; X: if a certain approach was not incorporated.

**Table 3:** Summary of risk thresholds and screening pathways (by cancer type).

Cancer (s)	Author (Year)	Risk Thresholds	Screening Pathway
Breast	Brooks (2021) [46]	3 risk categories based on 10-year/remaining lifetime risk	6 pathways based on 2 age categories and 3 risk levels
	Esserman (2017) [38]	3 risk categories based on 5-year risk	3 pathways based on age, risk levels and breast density
	Evans (2023) [43]	4 risk categories	No screening intervals specified; participants were followed at baseline, 3 months, and 6 months
	Laza-Vasquez (2022) [45]	3 risk categories	8 groups based on age and risk levels
	Liu (2022) [41]	2 risk categories	3 groups based on age and risk threshold levels
	Rainey (2022) [48]	4 risk categories	4 groups based on age and risk threshold levels
	Yen (2016) [42]	2 risk categories	3 groups based on risk threshold levels
Colorectal	Chen (2023) [34]	2 risk categories	2 groups based on risk threshold levels
	Emery (2023) [11]	2 risk categories	2 groups based on risk threshold levels
	Saya (2022) [40]	3 risk categories	2 groups based on risk threshold levels
	Schroy (2016) [35]	2 risk categories	No screening intervals specified
	Trevena (2022) [39]	3 risk categories	3 groups based on age, family history of cancer, and risk threshold levels
Prostate	Fredsøe (2020) [36]	3 risk categories	No screening intervals specified; participants were followed at 2-year
	Shah (2021) [47]	2 risk categories	7 groups based on age, PSA levels, and risk threshold levels
Lung	Field (2015) [37]	2 risk categories	5 groups based on nodule classifications
Ovary	Gaba (2020) [44]	3 risk categories	No screening intervals specified

**Table 4:** Key components of screening studies (by cancer type).

	Study Name (Registration No.)	Primary Outcome	Measurements	Main Results
Breast	PERSPECTIVE I&I ^1^ (Not found) [46]	Acceptability and feasibility of risk-based screening, uptake of genetic testing for risk assessment and screening behaviors.	Identification of new predisposition genes; Assessment of different recruitment and data-collection strategies using questionnaires; Assessment of acceptability and healthcare system readiness through survey and online forums; Economic analysis using administrative data.	Not yet available.
	WISDOM (NCT02620852) [38]	Safety (non-inferiority).	Rate of stage IIB cancers or higher diagnosed in annual vs. risk-based screening arms.	Not yet available (estimated completed date: 1 December 2024).
		Morbidity.	Rate of recall and breast biopsy between arms.	
	BC-Predict (NCT04359420) [43]	Feasibility.	Screening attendance at or within 180 days of the initial screening appointment; Uptake to BC-Predict of those attending screening; Time to provision of risk feedback letter and proportion over 8-week threshold; Subsequent consultation in clinics (telephone, as face-to-face not possible); Subsequent enrolment for more frequent mammography (NICE approved through clinics to age 60 or self-funded outside); Subsequent prescription of breast-cancer-preventive-medication.	Overall uptake of BC-Predict in screening attendees was 16.9%; 76.8% of those received risk feedback within the 8-week timeframe.
	DECIDO (NCT03791008) [45]	Attitude towards personalized breast cancer screening.	Attitude was measured with a three-item scale, each item ranging from 1 to 5, with higher scores indicating more positive attitudes. A “positive attitude” was defined as a total score greater than or equal to 12.	High positive attitude score towards personalized screening, with a median of 12. 2 out of 3 women scored > 12 indicting positive attitudes towards personalized screening; The intention to participate in personalized breast screening rated as “definitely will” or “likely to” by 9 out of 10 women; 97% of the women declared satisfied/very satisfied with personalized breast screening; 2.6% were not sure; 1 was very unsatisfied.
		Intention to participate in personalized breast cancer screening.	Intention to participate was measured with a 5-point Likert scale from definitely will (1) to definitely will not (5).	
		Satisfaction with personalized breast cancer screening.	Satisfaction was measured on a 5-point Likert scale from very unsatisfied to very satisfied.	
	BREATHE (Not found) ^2^ [41]	Acceptability, willingness, and cost-effectiveness.	Satisfaction survey	Not yet available.
	PROCAS (ISRCTN91372184) [48]	Key factors in participants’ adoption of screening and prevention recommendations after cancer risk communication.	Early detection behaviors: - Intent to request supplemental mammography outside the national screening programme (yes vs. no or do not know; for average and moderate risk groups); - Increased breast self-examination (yes vs. no). Preventive behaviors: - Started with preventative medication (yes vs. no; for moderate and high-risk groups); - Changed diet (yes vs. no); - Changed physical activity levels (yes vs. no); - Changed alcohol intake (yes vs. no).	Predictors of intent to request supplemental mammography outside the national screening program includes the following: - Supplemental screening and breast self-examination; - Risk-reducing medication; - Preventive lifestyle behaviors; - Having a first degree relative with breast cancer; - Higher age; - Higher body mass index.
	Not found ^3^ [42]	Effectiveness	Detection (diagnosis) rates; Stage II+ disease incidence; Mortality from breast cancer; Overdiagnosis	Cancer detection rates were highest for universal biennial mammography (4.9% and 3.0%, respectively), followed by risk-based mammography (2.8% and 2.8%, respectively), and lowest for annual CBE (0.97% and 0.70%, respectively). Universal biennial mammography screening, compared with annual CBE, was associated with a 41% mortality reduction (*** RR = 0.59; 95%CI, 0.48–0.73) and a 30% reduction in stage II+ breast cancer (*** RR = 0.70; 95%CI, 0.66–0.74). Risk-based mammography screening was associated with an 8%reduction in stage II+ breast cancer (*** RR = 0.92; 95%CI, 0.86–0.99) but was not associated with a statistically significant mortality reduction (*** RR = 0.86; 95%CI, 0.73–1.02). Estimates of overdiagnosis were no different from CBE for risk-based screening and 13% higher than CBE for universal mammography.
Colorectal	TARGET-C (ChiCTR1800015506) [34]	Detection rate of advanced colorectal neoplasms (CRC and advanced precancerous lesions)	Advanced adenoma was defined as adenoma with at least 1 of the following features: - High-grade dysplasia, - Villous or tubulovillous histologic features - Size ≥ 10 mm. Advanced serrated adenoma was defined as any serrated adenoma (traditional serrated adenoma or sessile serrated lesion) ≥10 mm or dysplasia; Both advanced adenoma and advanced serrated lesions were regarded as advanced precancerous lesions.	Colonoscopy (Arm 1): 2.76% * FIT (Arm 2): 2.17% Risk-adapted (Arm 3): 2.35% ** OR _colonoscopy vs. FIT_ = 1.27 (95% CI: 0.99–1.63) ** OR _colonoscopy vs. risk-adapted_ = 1.17 (95% CI:0.91–1.49) ** OR _risk-adapted vs. FIT_ = 1.09 (95% CI:0.88–1.35) Numbers of colonoscopies to detect 1 advanced neoplasm: 15.4, 7.8, and 10.2, respectively
	CRISP (ACTRN12616001573448) [11]	Proportion of participants who completed risk-appropriate CRC screening at 12-month follow-up	Screening participation rate was obtained from: Self-report; GP record audit; Medicare Benefits Schedule; NBCSP; Victorian Admitted Episodes Dataset (VAED).	Intervention vs. Control: 6.5% absolute increase (95%CI: –0.28–13.2); 20.3% increase in those due CRC screening during follow-up (95%CI: 10.3–30.4)
	SCRIPT (ACTRN12621000092897p) [40]	Impact of the SCRIPT intervention on risk-appropriate CRC screening after 12 months.	Difference between intervention and control arms in the proportion of participants who have had risk appropriate CRC screening at 12 months. follow-up.	Not available
	NCT01596582 [35]	Concordance between patient preference and test ordered.	Tracked using BMC’s electronic medical record ordering system.	No significant differences in concordance were observed.
	ACTRN12611000534987 [39]	Risk Appropriate Screening (RAS) and colorectal cancer screening uptake.	Through online CRC patient survey (24 screening and 9 patient demographic items).	The intervention significantly increased RAS in high-risk participants compared with UCG (80.0% vs. 64.0%, respectively; OR = 3.14, 95% CI: 1.25–7.96) but not in average-risk (44.9% vs. 49.5%, respectively; OR = 0.97, 95% CI: 0.99–1.12) or moderate-risk individuals (67.9% vs. 81.1%, respectively; OR = 0.40, 95% CI: 0.12–1.33).
Prostate	ProCaRis (NCT01739062) [36]	Proportion of men having a repeated PSA test within 2 years.	Proportion of men having a repeated PSA test within 2 years.	At 2 years after inclusion, a total of 1218 men (34.2%) in the intervention practices and 1628 (38.4%) men in the control practices had a PSA test (OR = 0.95, 95% CI 0.78–1.14, *p* = 0.56); Men of high genetic risk had a higher propensity for repeated PSA testing within 2 years than men of normal genetic risk (** OR = 8.94, *p* < 0.01).
	Not found ^4^ [47]	Evaluate the implementation of a risk-based prostate-cancer-screening algorithm.	Percent of men who met screening algorithm criteria; Percent of men with a PSA result.	Percent of men who met screening algorithm criteria: 49.3% (pre-implementation) vs. 68.0% (post-implementation) (*p* < 0.001); Total number of men with abnormal PSA: Pre-implementation *n* = 366, 2.7%; Post-implementation *n* = 583, 4.9%.
Lung	UKLS (ISRCTN 78513845) [37]	Effectiveness of risk prediction modeling; Evaluation of volumetric analysis in the management of CT-detected nodules; Cost-effectiveness.	Population-based recruitment based on risk stratification; Study management through a web-based database; Define optimal characteristics of CT readers (radiologists vs. radiographers); Characterization of CT-detected nodules utilizing volumetric analysis; Prevalence of lung cancer at baseline; Socio-demographic factors affecting participation; Psychosocial measures; Cost-effectiveness modeling.	In total, 42 participants (2.1%) had confirmed lung cancer, 34 (1.7%) at baseline and 8 (0.4%) at the 12-month scan; - 28/42 (66.7%) had stage I disease - 36/42 (85.7%) had stage I or II disease - 35/42 (83.3%) had surgical resection In total, 536 subjects had nodules greater than 50 mm^3^ or 5 mm diameter - 41/536 were found to have lung cancer. - 1 further cancer was detected by follow-up of nodules between 15 and 50 mm^3^ at 12 months. The baseline estimate for the incremental cost-effectiveness ratio of once-only CT screening, under the UKLS protocol, was £8466 per quality adjusted life year gained (CI £5542 to £12,569).
Ovary	PROMISE-FS (ISRCTN54246466) [44]	Acceptability.	Responses to the decision aid questions and overall score.	Decision aid satisfaction: 92.2%; Telephone helpline use rate:13% Questionnaire response rate at six months: 75%.
		Uptake of the study.	Number of individuals who express interest in participating in the study (by post/email/telephone).	

^1^ Reason for non-registration: This is a pre-implementation project rather than a clinical trial; ^2^ Reason for non-registration: This is a study testing personalized risk assessment rather than a clinical trial; ^3^ Reason for non-registration: This is a population-based analysis rather than a clinical trial; ^4^ Reason for non-registration: This is a quality improvement intervention rather than a clinical trial. * FIT: Fecal immunochemical test; ** OR: Odds ratio; *** RR: Risk ratio.

**Table 5:** Benefits–harm aummary for screening studies (by cancer type).

Cancer (s)	Study Name/No.	Benefits	Harms	Benefit-Harm Assessment
Breast	PERSPECTIVE I&I ^1^ [46]	Not available	Not available	Not available
	WISDOM [38]	Not available (estimated primary completed date: 1 December 2024)	Not available (estimated primary completed date: 1 December 2024)	Not available (estimated completed date: 1 December 2024)
	BC-Predict [43]	Risk stratification can be performed as part of routine NHS Breast Screening Programme delivery and supports the uptake of preventive medicines for women at high risk.	No evidence of adverse effects on anxiety beyond transient cancer worry; The practice of delivering risk-based screening was much less burdensome than healthcare professionals anticipated prior to Delivery.	Measures to increase uptake should be addressed, especially among women from lower socioeconomic and ethnic minority backgrounds.
	DECIDO [45]	Positive attitude towards personalized breast screening; expressed strong intention to participate; and very satisfied with having participated in the study.	Knowledge of the benefits and harms of breast screening was low, especially with regard to false positives and overdiagnosis.	Demonstrates the need to create tools and strategies for developing interventions that focus on raising awareness about personalized screening and increasing literacy in risk measurement.
	BREATHE ^2^ [41]	Only the study protocol is available.	Only the study protocol is available.	Only the study protocol is available.
	PROCAS [48]	Breast cancer risk communication predicts the uptake of key tailored primary and secondary preventive Behaviors.	Not reported	Effective communication of breast cancer risk information is essential to optimize the population wide impact of tailored screening.
	Not found ^3^ [42]	Compared with population-based screening for breast cancer with annual CBE, universal biennial mammography resulted in a substantial reduction in breast cancer deaths, whereas risk-based biennial mammography resulted in only a modest benefit.	Compared with annual CBE, risk-based and universal mammography screening did not result in significant overdiagnosis of breast cancer.	The findings should be informative to health policymakers seeking to determine if and how they might initiate breast-cancer-screening programs.
Colorectal	TARGET-C [34]	The risk-adapted approach saved 33% of endoscopy resources required for detecting 1 advanced neoplasm compared with the 1-time colonoscopy screening.	Costs for detecting 1 advanced neoplasm from the societal perspective were higher in the risk-adapted screening compared with the 1-time colonoscopy screening.	The risk-adapted approach is a feasible and cost-effective strategy for population-based CRC screening; The need for additional screening after 3 rounds or an expansion the screening interval should be addressed in future studies.
	CRISP [11]	A risk assessment and decision support tool increase risk-appropriate CRC screening in those due screening.	There were no differences between groups at any timepoint on general or cancer-specific anxiety or absolute risk perception.	Not reported
	SCRIPT [40]	Only the study protocol is available.	Only the study protocol is available.	Only the study protocol is available.
	NCT01596582 [35]	Concordance was positively associated with satisfaction with the decision-making process, screening intentions, and test completion rates.	Not specified	Providers perceived risk stratification to be useful in their decision-making but often failed to comply with patient preferences for tests other than colonoscopy.
	ACTRN12611000534987 [39]	Online CRC risk calculator increased risk appropriate screening in high-risk participants and improved screening uptake overall within a 12 m follow-up period.	Harms and costs of more invasive screening tests such as colonoscopy in lower risk individuals.	Online CRC risk calculator may be useful for facilitating the uptake of risk-based screening guidelines.
Prostate	ProCaRis [36]	Among participants who had a genetic test, knowledge of genetic risk significantly influenced future PSA testing.	Genetic test to assess lifetime risk of prostate cancer did not reduce the overall number of future PSA test.	The follow-up period of 2 years was not long enough to draw final conclusions about the effect of this intervention on the diagnosis or mortality rate of prostate cancer.
	Not found ^4^ [47]	The adjusted odds of meeting algorithm-based screening was 6.5 times higher in the post-implementation period than in the pre-implementation period.	Not reported	An increase in screening in higher-risk groups balanced with a decrease in screening in low-risk groups.
Lung	UKLS [37]	It is possible to detect lung cancer at an early stage and deliver potentially curative treatment in over 80% of cases; Health economic analysis suggests that the intervention would be cost effective.	False-positive rate: 3.6% Interval imaging rate: 23.2%	CT screening in the United Kingdom is possible using a risk prediction model that avoids the selection of people at very low risk who are unlikely to benefit, and a nodule management algorithm that effectively manages indeterminate CT findings, yet detects a high number of early-stage lung cancers.
Ovary	PROMISE-FS [44]	85.5–98.7% were satisfied with their decisions; Ovarian cancer related worry (*p* = 0.021) and general cancer risk perception (*p* = 0.015) decreased over 6 months.	There were no significant effects on overall depression (*p* = 0.30), anxiety (*p* = 0.10), quality of life (*p* = 0.99), or distress level (*p* = 0.26).	Population-based personalized ovarian cancer risk stratification is feasible and acceptable, has high satisfaction, reduces cancer worry/risk perception, and does not negatively impact psychological health/quality of life.

^1^ Reason for non-registration: This is a pre-implementation project rather than a clinical trial; ^2^ Reason for non-registration: This is a study testing personalized risk assessment rather than a clinical trial; ^3^ Reason for non-registration: This is a population-based analysis rather than a clinical trial; ^4^ Reason for non-registration: This is a quality improvement intervention rather than a clinical trial.

## Data Availability

The data that support the study findings are accessible from the corresponding author upon request.

## Supplementary Materials

The following supporting information can be downloaded at: https://www.mdpi.com/article/10.3390/jpm14080863/s1, Table S1: Search Terms per Database; Table S2: Quality Assessment; Table S3: Reasons for Exclusion.
