# Supplementary material for: Targeted Screening for Cancer: Learnings and Applicability to Melanoma: A Scoping Review
Source: J Pers Med. 2024 Aug 14;14(8):863. doi: 10.3390/jpm14080863 (PMC11355139; doi:10.3390/jpm14080863)
Supplement: Supplementary file 1 [file jpm-14-00863-s001.zip › Supplementary material-Table S1-Search Terms per Database.pdf]

Table S1: Search Terms per Database

Scopus:

(TITLE-ABS-KEY (cancer) AND TITLE-ABS-KEY (screening) AND TITLE-ABS-KEY ((risk W/3 (tailor* OR personal*)) OR stratifi*)) AND PUBYEAR > 2012 AND (LIMIT-TO (LANGUAGE , "English")) AND (LIMIT-TO (EXACTKEYWORD , "Human"))

Cochrane via Ovid:

Set	Search Statement
1.	risk-based.mp.
2.	risk-tailor*.mp.
3.	Risk stratified.mp.
4.	"Early Detection of Cancer"/
5.	1 or 2 or 3
6.	4 and 5
7.	Limit 6 to (yr="2013-2023" and english language and humans)

Embase:

Set	Search Statement
1.	cancer screening/
2.	risk-based.mp.
3.	risk-tailor*.mp.
4.	Risk stratified.mp.
5.	2 or 3 or 4
6.	1 and 5
7.	Limit 6 to (yr="2013-2023" and english language and humans)

Medline:

Set	Search Statement
1.	risk-based.mp.
2.	risk-tailor*.mp.
3.	risk stratified.mp.
4.	"Early Detection of Cancer"/
5.	1 or 2 or 3
6.	1 and 5
7.	Limit 6 to (yr="2013-2023" and english language and humans)

Psychinfo:

Set	Search Statement
1.	Exp Cancer Screening/
2.	risk-based.mp.
3.	risk-tailor*.mp.
4.	risk stratify*.mp.
5.	2 or 3 or 4
6.	1 and 5
7.	Limit 6 to (yr="2013-2023" and english language and humans)

CINAHL:

Set	Search Statement
1.	"cancer Screening"
2.	"risk-based"
3.	"risk stratifi"
4.	"risk-tailor"

5.	2 or 3 or 4
6.	1 and 5
7.	Limit 6 to (yr="2013-2023" and english language and humans)
