# Supplementary material for: Targeted Screening for Cancer: Learnings and Applicability to Melanoma: A Scoping Review
Source: J Pers Med. 2024 Aug 14;14(8):863. doi: 10.3390/jpm14080863 (PMC11355139; doi:10.3390/jpm14080863)
Supplement: Supplementary file 1 [file jpm-14-00863-s001.zip › Supplementary material-Table S2-Quality Assessment.pdf]

Table S2: Quality Assessment-non-RCT studies

	Author (year)	Confounding Bias	Selection Bias	Bias in classification of interventions	Bias due to deviations from intended interventions	Bias due to missing data	Bias in measurement of outcomes	Bias in selection of the reported result	Overall
1	Brooks (2021)	Low Risk	Low Risk	Low Risk	No information	Low Risk	Low Risk	Low Risk	Good Quality
2	Evans (2023)	Low Risk	Low Risk	Low Risk	No information	Low Risk	Low Risk	Low Risk	Good Quality
3	Laza-Vasquez (2022)	Low Risk	Low Risk	Low Risk	No information	Low Risk	Low Risk	Low Risk	Good Quality
4	Liu (2022)	Low Risk	Low Risk	Low Risk	No information	No information	Low Risk	No information	Good Quality
5	Rainey (2022)	Low Risk	Low Risk	Low Risk	No information	Low Risk	Low Risk	Low Risk	Good Quality
6	Yen (2016)	Low Risk	Low Risk	Low Risk	Low Risk	Low Risk	Low Risk	Low Risk	Good Quality
7	Shah (2021)	Low Risk	Low Risk	Low Risk	Low Risk	Low Risk	Low Risk	Low Risk	Good Quality
8	Gaba (2020)	Low Risk	Low Risk	Low Risk	No information	Low Risk	Low Risk	Low Risk	Good Quality

Table S2: Quality Assessment-RCT studies

	Author (year)	Risk of bias (randomization)	Risk of bias (deviations from intended interventions)	Missing outcome data	Risk of bias (outcome measurement)	Risk of bias (reported result)	Overall
9	Esserman (2017)	Low Risk	Low Risk	No information	Low Risk	No information	Good Quality
10	Chen (2023)	Low Risk	Low Risk	Low Risk	Low Risk	Low Risk	Good Quality
11	Emery (2023)	Low Risk	Low Risk	Low Risk	Low Risk	Low Risk	Good Quality
12	Saya (2022)	Low Risk	Low Risk	No information	Low Risk	No information	Good Quality
13	Schroy (2016)	Low Risk	Low Risk	Low Risk	Low Risk	Low Risk	Good Quality
14	Trevena (2022)	Low Risk	Low Risk	Low Risk	Low Risk	Low Risk	Good Quality
15	Fredsøe (2020)	Low Risk	Low Risk	Low Risk	Low Risk	Low Risk	Good Quality
16	Field (2015)	Low Risk	Low Risk	Low Risk	Low Risk	Low Risk	Good Quality
