# Supplementary material for: Targeted Screening for Cancer: Learnings and Applicability to Melanoma: A Scoping Review
Source: J Pers Med. 2024 Aug 14;14(8):863. doi: 10.3390/jpm14080863 (PMC11355139; doi:10.3390/jpm14080863)
Supplement: Supplementary file 1 [file jpm-14-00863-s001.zip › Supplementary material-Table S3-Reasons for Exclusion.pdf]

Table S3: Reasons for Exclusion

Reference		Reason for exclusion	Reasons presented in PRISMA diagram
1	The UK lung cancer screening trial: A pilot randomised controlled trial of low-dose computed tomography screening for the early detection of lung cancer	Participants should come from the adult general population; however, this study targeted individuals with high risk of developing lung cancer	Wrong participants
2	Risk-adapted screening for bladder cancer: Daily routine in preventive care with the assessment tool RiskCheck Bladder Cancer© in the urological health-service research association IQUO, Germany	Participants should come from the adult general population; however, this study targeted asymptomatic patients	Wrong participants
3	Risk prediction of cervical abnormalities: The value of sociodemographic and lifestyle factors in addition to HPV status	Not a risk-based screening study, but rather assessing risk factors other than HPV status	Wrong study design
4	Comparing U.S. Preventive Services Task Force 2013 versus 2021 lung cancer screening eligibility	Not risk-based screening study, but a comparison between two guidelines (USPSTF 2021 VS USPS TF 2013)	Wrong study design
5	Risk stratification for melanoma	Editorial/letter/comment/opinion	Editorial/letter/comment/opinion
6	My PeBS (My Personal Breast Screening): A European clinical study evaluating a personalised risk-based screening strategy	No access to full text	No access to full text
7	Prostate-specific antigen (PSA) levels in men 60 to 70 years of age predict aggressive prostate cancer in the PLCO cancer screening trial: Implications for risk-stratified screening	No access to full text	No access to full text
8	Risk-based breast cancer follow-up stratified by age	Not a risk-based screening study, but a model testing study (POMDP: partially observable Markov decision process)	Wrong study design
9	Risk stratification of HPV 16 DNA methylation combined with E6 oncoprotein in cervical cancer screening: A 10-year prospective cohort study	Not a risk-based screening study, but rather testing the use of standalone HPV 16 methylation or its combination with E6 oncoprotein for cervical cancer screening	Wrong study design
10	Development and pilot of an online, personalized risk assessment tool for a breast cancer precision medicine trial	Not a risk-based screening study;<br>Wrong study design: mixed method study;<br>Participants should come from the adult general population; however, this study targeted women with elevate risk for breast cancer	Wrong study design
11	Prostate cancer screening using a combination of risk-prediction, MRI, and targeted prostate biopsies (STHLM3-MRI): a prospective, population-based, randomised, open-label, non-inferiority trial	Participants should come from the adult general population; however, this study targeted men aged 50-74 years with an elevated risk of prostate cancer	Wrong participants
12	Ethical, Legal, and Regulatory Issues for the Implementation of Omics-Based Risk Prediction of Women's Cancer: Points to Consider	Review	Review

13	The WISDOM study pilot: Evaluating a preference-tolerant RCT of risk-based vs. annual breast cancer screening	No access to full text	No access to full text
14	Prostate cancer risk assessment by the primary care physician and urologist: transabdominal- versus transrectal ultrasound prostate volume-based use of the Rotterdam Prostate Cancer Risk Calculator	Not a cancer screening study, but rather testing the accuracy of transabdominal ultrasound (TAUS) measured prostate volume in the primary care setting	Wrong study design
15	"It Will Lead You to Make Better Decisions about Your Health"—A Focus Group and Survey Study on Women's Attitudes towards Risk-Based Breast Cancer Screening and Personalised Risk Assessments	Not a cancer screening study, but rather focus group discussions and surveys.	Wrong study design
16	Diagnostic yield using a fit-based risk model was not better than fit only: a randomized controlled trial in the second round of the Dutch colorectal cancer screening program	No access to full text	No access to full text
17	Performance of a simplified scoring system for risk stratification in oral cancer and oral potentially malignant disorders screening	No access to full text	No access to full text
18	Bladder cancer risk stratification using a urinary mRNA biomarker panel – A path towards cystoscopy triaging	No access to full text	No access to full text
19	Mypebs: An international randomized study comparing personalized, risk-stratified to standard breast cancer screening in women aged 40-70	No access to full text	No access to full text
20	Increasing uptake to a lung cancer screening programme: building with communities through co-design	Not risk-based screening study, but interviews and focus groups;<br>Participants should come from the adult general population; however, this study recruited those at high risk from developing lung cancer and professionals who may provide or signpost to a future lung cancer screening programme	Wrong study design
21	Expanding the boundaries of previously obtained informed consent in research: Views from participants in the Personalised Risk-based Mammascreening study	Not a cancer screening study, but a qualitative study (focus group discussion)	Wrong study design
22	Personalized Risk-Based Screening Design for Comparative Two-Arm Group Sequential Clinical Trials	Review	Review
23	Potential of using mammography screening appointments to identify high-risk women: cross sectional survey results from the national health interview survey	Not a cancer screening study, but a study to identify high-risk women for breast cancer by estimating the (1) proportion of high-risk women who report that they have undergone mammographic screening and the (2) proportion of high-risk women who receive recommendations for breast MRI screening	Wrong study design

24	Tailoring screening to individual risk decreases the cost and improves the value of screening	No access to full text	No access to full text
25	"It's personalized, but it's still bucket based": the promise of personalized medicine vs. the reality of genomic risk stratification in a breast cancer screening trial	Not a risk-based screening study, but rather findings presented from a previous innovative trial design	Wrong study design
26	Uptake of Risk Appropriate Behaviors After Breast Cancer Risk Stratification in the Mammography Screening Population	Not risk-based screening, but a case study	Wrong study design
27	Diagnostic yield using a fit-based risk model was not better than fit only: A randomized controlled trial in the second round of the Dutch colorectal cancer screening programme	No access to full text	No access to full text
28	Cervical cancer screening research in the PROSPR I consortium: Rationale, methods and baseline findings from a US cohort	Not a risk-based screening study, but rather a description of rationale, methods, and baseline findings from a US cohort from cervical cancer screening research	Wrong study design
29	Distribution of estimated lifetime breast cancer risk among women undergoing screening mammography	Not a risk-based screening study, but rather investigating the concordance of three breast cancer risk prediction models	Wrong study design
30	Attitudes towards a programme of risk assessment and stratified management for ovarian cancer: A focus group study of UK South Asians' perspectives	Not a risk-based screening study, but a focus group discussion	Wrong study design
31	Management of Lung Cancer Screening Results Based on Individual Prediction of Current and Future Lung Cancer Risks	Not a risk-based screening study, but testing LCRAT+CT_{pos} Model in lung cancer	Wrong study design
32	Randomized controlled trial of storytelling compared to a personal risk tool intervention on colorectal cancer screening in low-income patients	Participants should come from the adult general population; however, this study recruited low-income patients	Wrong participants
33	Lung cancer screening: Enhancing risk stratification and minimising harms by incorporating information from screening results	Editorial/letter/comment/opinion	Editorial/letter/comment/opinion
34	Shifting risk-stratified early prostate cancer detection to a primary healthcare setting	Not a risk-based screening study, but a comparison between multivariable risk stratification and current referral indicator (PSA threshold of 3.0 ng/mL)	Wrong study design
35	Exploring a novel method for optimising the implementation of a colorectal cancer risk prediction tool into primary care: a qualitative study	Not a cancer screening study, but a qualitative study	Wrong study design
36	Increasing referral of at-risk women for genetic counseling and BRCA testing using a screening tool in a community breast imaging center	Not a cancer screening study, but rather evaluating the feasibility of using a screening tool at a breast imaging center to increase HBOC assessment referrals	Wrong study design
37	Real-time genotyping-based breast cancer risk assessment in MyPeBS, an international randomized trial in the general	No access to full text	No access to full text

	population comparing risk-stratified to standard breast cancer screening (BCS)		
38	The ANDROMEDA prospective cohort study: Predictive value of combined criteria to tailor breast cancer screening and new opportunities from circulating markers: Study protocol	Not a cancer screening study, but rather evaluating the diagnostic accuracy of selected circulating microRNAs	Wrong study design
39	Risk Stratification and Shared Decision Making for Colorectal Cancer Screening	Participants should come from the adult general population; however, this study targeted asymptomatic, average-risk patients	Wrong participants
40	Personalized risk assessment for prevention and early detection of breast cancer: Integration and implementation (PERSPECTIVE I&I)	Editorial/letter/comment/opinion	Editorial/letter/comment/opinion
41	Economic evidence for the use of risk-selection and risk-stratification for lung cancer screening programs	No access to full text	No access to full text
42	Counselling in the population genomics context: Participant experiences in the wisdom breast cancer screening trial	Editorial/letter/comment/opinion	Editorial/letter/comment/opinion
43	Biomarkers in melanoma and non-melanoma skin cancer prevention and risk stratification	Not a risk-based screening study, but rather a discussion of biomarkers for melanoma and non-melanoma skin cancers	Wrong study design
44	Risk-tailored starting age of breast cancer screening based on women's reproductive profile: A nationwide cohort study	Not a risk-based screening study, but a retrospective data analysis using nationwide, registered-based Swedish datasets	Wrong study design
45	Implementation of an EHR-based risk-stratified prostate cancer screening program in a primary care network	No access to full text	No access to full text
46	Cost-Effectiveness Analysis of HPV Extended versus Partial Genotyping for Cervical Cancer Screening in Singapore	Not a risk-based screening study, but rather a cost-effectiveness analysis of HPV Extended versus Partial Genotyping for Cervical Cancer Screening	Wrong study design
47	Health professionals' perspectives on breast cancer risk stratification: Understanding evaluation of risk versus screening for disease	Not a cancer screening study, this study explores health professionals' perspectives on breast cancer risk stratification	Wrong study design
48	The Lungscreen WA project: Feasibility of LDCT screening with the PLCOM2012 risk model and PanCan nodule risk calculator	No access to full text	No access to full text
49	EE442 The Cost-Effectiveness of Risk Stratified Breast Cancer Screening in the UK	No access to full text	No access to full text
